# Meta-Analysis on the Prevalence of Failed Transfer of Passive Immunity in Calves from Pasture-Based Dairy Farms in Australasia

**DOI:** 10.3390/ani13111792

**Published:** 2023-05-28

**Authors:** Thien D. Van, Do T. Hue, Cynthia D. K. Bottema, Gebremeskel Mamu Weird, Rebel Skirving, Kiro R. Petrovski

**Affiliations:** 1 Davies Livestock Research Centre, School of Animal & Veterinary Sciences, University of Adelaide, Roseworthy Campus, Roseworthy, SA 5371, Australia; thien.vandinh@adelaide.edu.au (T.D.V.);; 2 Faculty of Animal Science, Vietnam National University of Agriculture, Trau Quy, Gia Lam, Hanoi 12406, Vietnam; 3 Gambier Vets Pty. Ltd., Mount Gambier, SA 5290, Australia; 4 Australian Centre for Antimicrobial Resistance Ecology, School of Animal & Veterinary Sciences, University of Adelaide, Roseworthy Campus, Roseworthy, SA 5371, Australia

**Keywords:** bovine, IgG, failure of passive transfer, colostrum, FPT, FPIT, passive immunity

## Abstract

**Simple Summary:**

The dairy industry in Australia and New Zealand is pasture-based, therefore, monitoring failed transfer of passive immunity (FTPI) is challenging. This study involved a systematic literature search and meta-analysis of papers reporting FTPI data from dairy calves within eight days of birth in Australia and New Zealand, until the end of 2022. An overall prevalence of 33% of FTPI in dairy calves in Australasia and a prevalence of 38% at the farm level were calculated from the meta-analysis, comparable to rates observed elsewhere. Factors, such as the frequency of calf removal from the calving area, time of the first colostrum feed after birth, colostrum volume and quality, and other management practices, were found to play a significant role in FTPI in Australasia.

**Abstract:**

Monitoring and minimizing the prevalence of failed transfer of passive immunity (FTPI) in dairy replacement calves within the first week of life is crucial for calf health and farm profitability. In this study, a systematic literature search and meta-analysis were conducted on papers reporting the prevalence of FTPI in calves from pasture-based dairy farms in Australia and New Zealand. Two search methods, a “traditional method” and a “search engine method”, were conducted to identify published studies on FTPI in Australia and New Zealand. Data from a total of 13,430 calves from eight studies in Australasia were included in the analysis for FTPI within 8 days of birth. The meta-analysis revealed that the average prevalence of FTPI was 33% across the two countries, with the lowest FTPI (9%) in Western Australia and the highest FTPI (59%) in New Zealand. Using farm data from three studies, the average prevalence of FTPI at the farm level in Australasia was 38%, with the lowest prevalence found in a farm in South Australia (6%). In conclusion, the meta-analysis confirmed the need for good management of cows and newborn calves after birth in pasture-based systems to reduce FTPI in calves. Collecting newborn calves from pasture at least twice per day after birth and providing colostrum of sufficient quantity and quality as soon as possible were the best practices for preventing FTPI in Australasian dairy systems.

## 1. Introduction

The dairy cattle industries in Australia and New Zealand are relatively unique with mainly pastoral management. The dairy sector is one of the most important rural industries for both Australasian countries, not only in terms of employment, but also in terms of production, with over 20 billion litres of milk produced annually for the domestic and international markets. In 2022, the total number of dairy cattle in Australia was 2.1 million head [1] and in New Zealand was 4.8 million head [2]. Australia remains a significant exporter of dairy products, and is ranked fourth in terms of world dairy trade with a 5% share, behind New Zealand (with approximately 20% of the global trade), the European Union and the United States [3].

One of the major concerns for the dairy cattle industry globally is the improvement of calf health within a few days of life. Calves are born agammaglobulinemic [4,5], and must rely on colostrum ingestion to obtain nutrients, growth factors, and immunoglobulins, mainly immunoglobulin G (IgG), for passive immunity [6,7]. The transfer of immunoglobulins from colostrum to newborn calf blood has been investigated extensively using different technical terms including “passive transfer of immunity”, and more recently, “transfer of passive immunity” [8]. The current term “transfer of passive immunity” (TPI) is more accurate as it is the immunity from colostrum ingestion that is passive rather than immunoglobulins being passively transferred, and TPI is the term used herein.

Calves should receive colostrum as soon as possible after birth and before ‘gut closure’, as gut closure prevents absorption of large molecules, including immunoglobulins, thereafter. Calf gut closure occurs approximately 24 h after birth, although this timing can vary [6,9]. If calves receive insufficient quality or quantity of colostrum prior to gut closure, this may result in failed transfer of passive immunity (FTPI)**,** and FTPI is correlated with increased calf morbidity and mortality [10,11,12,13]. Factors that affect the successful TPI in calves include the colostrum quantity and quality, timing of feeding colostrum, age and parity of the dam, and presence of the dam [4,14,15,16,17].

FTPI is diagnosed when the calf serum IgG concentration or total protein in the first few days of life is below specified thresholds. The serum IgG and total protein can be reliably tested for the transfer of passive immunity up to 9 days of age [18]. Measuring IgG in the calf serum after colostrum ingestion by radial immunodiffusion assay (RID) is the standard method for confirming TPI [6]. Many other methods for serum IgG measurement have been also developed though, including turbidimetric immunoassays and enzyme-linked immunosorbent assays [4,6]. An indirect method to identify FTPI in calves is to measure serum total protein, and this approach is widely accepted because the total serum protein concentration is strongly correlated with IgG levels [19,20,21]. Some common techniques for determining FTPI in newborn calves include Bradford assays, biuret methods, electrophoresis, serum refractometry and Brix refractometry [21,22]. The FTPI thresholds for dairy calves vary depending on the study and the method, however, the generally accepted thresholds for FTPI up to 9 days of age are <10 g/L of serum IgG [6,18,23,24,25], <50 to 52 g/L of serum total protein (TP) [25], and <8.1–8.5 Brix % [6,18,26]. Other thresholds for FTPI confirmation have been proposed for other indirect measurements, including serum/plasma gamma-glutamyl transferase (GGT) [6,25,27]. However, there is an ongoing debate about the level of specific FTPI thresholds using GGT [24,28,29].

Avoiding FTPI and ensuring good calf health are important to ensure profitability for dairy cattle producers. Hence, monitoring and minimizing the prevalence of FTPI in calves is essential to dairy production systems. Calves with FTPI have been shown to cause economic losses of around EUR 60/dairy calf with FTPI (95% CI = EUR 10–EUR109) in European cattle farms [30]. Unfortunately, in Australasia, monitoring for FTPI is rudimentary, with only 13% of surveyed dairy farms in Australia reporting evaluation [31]. In a New Zealand-based study, 76% of respondents thought that FTPI could increase the risk of calf morbidity and affect their future productivity, but only 1% of surveyed farmers reported routine monitoring for FTPI [17].

The uniqueness of Australasian dairy systems and the lack of FTPI monitoring led to this current study with two main objectives: (1) a systematic search and review of all published studies on FTPI in dairy calves born in pasture-based systems in Australasia (Australia and New Zealand) and (2) a meta-analysis of the prevalence of the FTPI rate in Australasia. The meta-analysis of FTPI prevalence overall was conducted using all relevant studies identified in the review and at the farm level using the studies reporting individual farm data.

## 2. Materials and Methods

### 2.1. Literature Search and Review

A systematic search of the literature on FTPI in dairy calves in Australia and New Zealand published between 1900 and December 2022, was conducted. To identify all published papers on FTPI in Australasia, two search methods, “traditional method” and “search engine method”, were used. Both search methods were conducted independently by three researchers (TDV, DTH, and KRP) using the same key words (Table 1). The searches were conducted, and websites accessed between January and February 2023.

The “traditional method” was conducted by one of the investigators (TDV) in Google Scholar (https://scholar.google.com.au/, accessed on 29 January 2023 to 26 February 2023) and the University of Adelaide library websites (https://www.adelaide.edu.au/library/, accessed on 29 January 2023 to 26 February 2023) using a general search function with the keywords (Table 1). In this traditional search, the researcher searched these keywords using the University of Adelaide library website without using a university account. Publication titles were scanned for relevance to TPI, and the list of relevant articles was created in a Microsoft Excel file.

**Figure 1:**
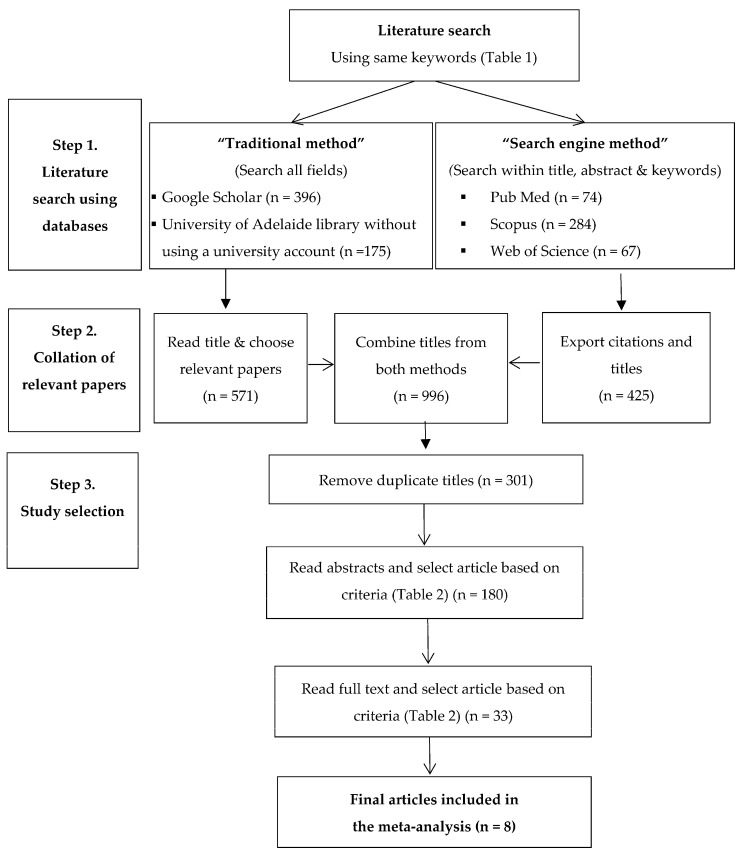
Steps in the systematic search, review, and meta-analysis of studies reporting prevalence of failed transfer of passive immunity in dairy calves in Australasian studies.

Two investigators (DTH and KRP) also used the “search engine method” to search for all studies in three different databases (Pub Med, Scopus, and Web of Science) using the same keywords as the “traditional method” search (Table 1). Investigators accessed these databases via a registered account that was purchased via the University of Adelaide library, and used an advanced search as following links:

PubMed: https://pubmed.ncbi.nlm.nih.gov/?otool=iauualib, accessed on 26 February 2023; 

Scopus: https://www.scopus.com/search/form.uri?display=basic#basic, accessed on 25 February 2023; 

Web of Science: https://www.webofscience.com/wos/woscc/basic-search, accessed on 24 February 2023;

The advanced search was used with “search engine method” in these three databases, where the above keywords (Table 1) were searched within “article title, abstract and keywords” only. Citations and abstracts of “search engine method” were exported for later review. The titles of articles were also exported into a separate Microsoft Excel file. Then, the titles from both search methods were combined to create a new dataset, and duplicates were removed (Figure 1).

The last step of the systematic search was article selection. Two investigators (TDV and DTH) independently read the title and abstract of the selected articles, and based on the selection criteria, decided whether to retain the article for further analysis (Table 2). For example, studies that investigated the effects of colostrum quality, volume, time, feeding program, vaccine, etc., on TPI or immunoglobulin G in calves, were not included in the dataset, but were considered for the review. Articles accepted by the two investigators were retained for further analysis, while those accepted by only one investigator were forwarded to a third investigator (KRP) for a decision.

All three investigators (TDV, DTH, KRP) read the full text of the selected articles to decide if the study met all the selection criteria (Table 2). All data were extracted from the remaining articles into a Microsoft Excel file. There were slightly different thresholds for estimating FTPI in calves between some studies for total serum protein, and studies using thresholds between the range of 50 to 52 g/L serum total protein were accepted.

### 2.2. Meta-Analysis

After the data from the papers were collected, selected, and extracted, they were organised into a spreadsheet. To ensure data quality, the data were further examined for errors, outliers, or missing values. If any issues were identified, the data were not included in the dataset. Descriptive data obtained from each article included the number of calves, age of calves when sampled, number of farms, thresholds for FTPI, and prevalence of FTPI.

The meta-analysis was performed in RStudio using the meta package [32]. The study weights were computed using the “Inverse” method, and the proportion data were transformed with the Freeman–Tukey double arcsine transformation method. A random-effects model was utilised to estimate the pooled effects. The standard errors and confidence intervals were estimated using the Hartung–Knapp method, and the DerSimonian–Laird estimator was used to calculate the between-study variance. The analysed data were then visualized using a forest plot [32].

The overall prevalence of FTPI in dairy calves in Australasia was estimated by using the prevalence FTPI in calves from the 8 selected studies (animal level) in the meta-analysis. In addition to using prevalence data of each study, the prevalence of FTPI in calves in Australasia at farm level was estimated using available farm data (*n* = 24 farms) from 3 studies (Lawrence et al. [33], Mason et al. [34] and Skirving et al. [16]) (referred herein as “prevalence of FTPI in calves at farm level”).

## 3. Results

### 3.1. Systematic Search and Review Results

By consolidating the search results from multiple sources and eliminating duplicates, 301 relevant articles were identified based on their titles (Figure 1, Table 3). After applying the selection criteria (Table 2), a total of eight articles addressing the prevalence of FTPI in dairy calves from 0–8 days after birth in Australian and New Zealand pasture-based dairy systems were identified and chosen for the meta-analysis (Table 4).

### 3.2. Meta-Analysis on the Prevalence of Failed Transfer of Passive Immunity in Australasia

#### 3.2.1. Failed Transfer of Passive Immunity in Australasia Overall

**Figure 2:**
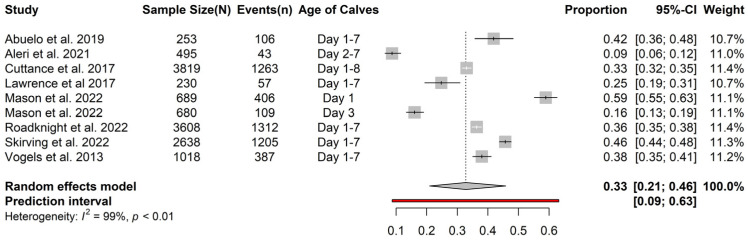
Overall prevalence of failed transfer of passive immunity in calves within 8 days of birth in Australasia determined from eight studies (total calves = 13,430) [16,31,33,34,35,36,37,38].

Data from a total of 13,430 calves from eight studies were used to calculate the overall prevalence of FTPI. The average prevalence of FTPI in calves within 8 days of birth in Australasia was 33% (95% CI = 21–46%) (Figure 2). The lowest prevalence of FTPI was reported in a study by Aleri et al. [35] in Western Australia (9%; 95% CI = 6–12%). The highest prevalence of FTPI in calves was reported in a study by Mason et al. [34] conducted in New Zealand on a total of 689 calves, sampled at day 1 of age (59%; 95% CI = 55–63%) (Figure 2).

#### 3.2.2. Failed Transfer of Passive Immunity in Australasia Based on Farm Data

**Figure 3:**
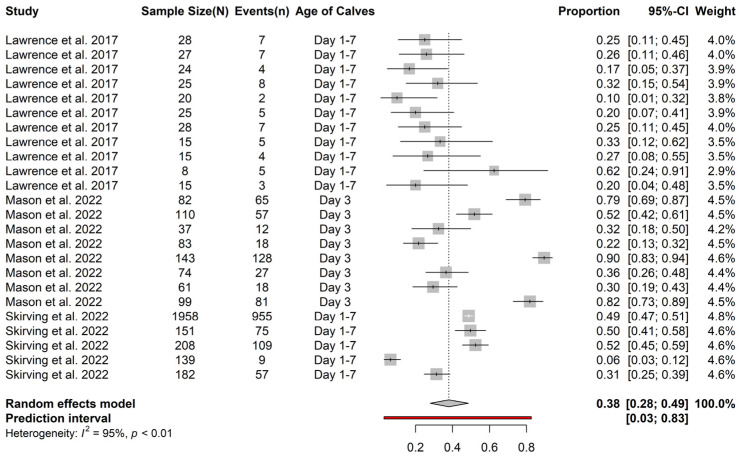
Prevalence of failed transfer of passive immunity in calves within 8 days of birth in Australasia using individual farm data (total calves = 3557) [16,33,34].

Farm data from three studies were used for a farm level meta-analysis (Figure 3), specifically, the studies by Lawrence et al. [33] (total farms = 11), Mason et al. [34] (total farms = 8), and Skirving et al. [16] (total farms = 5). The meta-analysis at the farm level indicated the prevalence of FTPI as 38% in dairy calves (95% CI = 28–49%). Only one farm in Australia (farm number 4) from Skirving et al. [16] had a very low proportion of FTPI (6%; 95% CI = 3–12%). The authors mentioned that on this farm, the newborn calves were collected twice per day, and the calves were fed 2–3 L of colostrum of a high quality (>22 Brix %) within 12 h of arriving in the shed [16].

## 4. Discussion

In this study, the literature on the prevalence of FTPI in calves within 8 days of birth in Australasia was systematically reviewed, and the data from selected articles were used in a meta-analysis. The results from the meta-analysis of the selected articles indicated the overall prevalence of FTPI in Australasian dairy calves was 33% (95% CI = 21–46%) and was slightly higher if individual farm data were used (38%; 95% CI = 28–49%).

### 4.1. Systematic Search Methods

In recent years, systematic searches and reviews of the literature have become more efficient and accessible due to the significant advancements in the tools designed for searching published articles. As a result, we were able to quickly locate all studies pertaining to the transfer of passive immunity from worldwide journals across an extensive time frame using various online databases. Herein, two search methods were employed to find relevant studies for the review and meta-analysis: the “search engine method” and the “traditional search” method. The “search engine method” involved searching keywords within the “abstract, title and keywords” in three major online databases (Scopus, PubMed, and Web of Science) using a registered account, while the “traditional search method” involved searching all information online using the same keywords. The “search engine” approach proved more effective and faster that the “traditional search method” for several reasons. Firstly, by focusing on keywords in the “abstract, title and keywords”, the search results were narrower, making it easier to export all abstracts and citations to Endnote and Microsoft Excel files for further screening. Secondly, Scopus, the largest database of citations and abstracts launched by Elsevier, provided the greatest number of relevant studies (*n* = 284). These findings supported the efficacy of Scopus and time-saving capabilities for systematic reviews observed previously [39]. Third, using multiple databases in conjunction with Scopus, including PubMed and Web of Science, allowed for a comprehensive search and collection of studies and citations, saving time during the initial search step.

In contrast, the “traditional search” method, which relied on Google Scholar and the University library website without an account, was found to be less effective and more time-consuming. This was due to the sheer volume of studies retrieved, requiring researchers to scan article titles before uploading citations. Additionally, duplicate articles and irrelevant sources, such as proceedings, theses, and newsletter articles, were encountered and required manual removal, further delaying the review process.

### 4.2. Meta-Analysis on FTPI in Australia and New Zealand

The overall prevalence of FTPI in Australasian calves was 33% (animal level), and 38% (farm level). These values were in the range of FTPI prevalence found in studies conducted elsewhere. For example, in a review by de Souza et al. [22], the prevalence of FTPI reported in Canada was 37–43% and in Italy was 41%. In another recent review, the prevalence of FTPI in veal calves was noted to be 40% in Belgium and 62.5% in Québec [40]. On the other hand, the prevalence of FTPI in calves was reported in the U.S.A. to have decreased from 40% in 1991–1992, to 20% in 2007, and to 12% by 2014 (also reviewed by de Souza et al. [22]). The observed differences in FTPI prevalence in calves across countries and times can be attributed to variation in management practices, environmental conditions, sample size and selection, and the timing of studies.

If farms are in the same geographical location, then management differences would be presumably the main factor affecting the prevalence of FTPI. Although dairy producers are aware of the effects of FTPI in calves, plans to monitor for FTPI in their calves do not appear to be a priority on many farms in Australasia [31]. Only 13% of surveyed dairy farmers in Australia reported undertaking routine assessment of FTPI on their farms [31]. Several factors may affect the decision not to measure FTPI, for example, producers may not fully understand the long-term impacts of FTPI or may need to prioritize other farm management tasks over routine FTPI monitoring. The net result though, is that there is reduced implementation of farm plans targeting the prevention of FTPI.

The threshold of 10 g/L of IgG was nearly uniformly accepted. However, the thresholds for serum total protein differed, and ranged from 50 to 52 g/L for the eight selected articles (Table 4), which could lead to biases. For example, the prevalence of FTPI in calves was 40%, 33%, 27% and 22%, when the corresponding serum total protein thresholds were 55, 52, 50, and 48 g/L, respectively [36]. A consensus threshold for serum total protein would be welcome as it would assist in clearly defining FPTI prevalence using this measure. However, standardising the threshold for Brix % would be even more valuable, as Brix refractometry can be used as an on-farm measure of FTPI [6,22].

### 4.3. Management Practices to Reduce FTPI in Australia and New Zealand

Factors associated with improved serum IgG levels in newborn calves include the quality, quantity, and bacterial content of the colostrum and the feeding time after birth [6,41]. Indeed, the management of FPTI is easier on farms with a small number of calves born at a given time and/or where calves are housed. However, the dairy cattle industry in Australasia is unique with calving occurring at pasture and calves normally being collected once or twice per day from the calving paddock. Many calves have suckled an unknown amount of colostrum from their dams before being collected.

#### 4.3.1. Timing of First Colostrum Feeding

Most farmers in Australia and New Zealand separate calves from their dams within 12–24 h following birth. To ensure calves receive adequate passive immunity, farmers provide the calves with colostrum, but the colostrum is pooled and often includes transitional milk [16,38]. In a study conducted in Victoria, Australia, 53% and 27% of total farms removed newborn calves 1–2 times/day, respectively, and only 5% of farms removed 3–4 times/day [38]. The overall prevalence of FTPI in this study was 25%. The relatively high prevalence of FTPI in calves may be a result of the late separation of calves from their dams and the calf unable to suckle enough colostrum from their dam or the colostrum from an individual dam may be of poor quality or quantity [31,38]. In another study conducted on 23 farms Australia-wide, 24% of farmers let the calves suckle colostrum from their dams after birth, and 72% of farmers separated calves from their dams >6 h after calving [31]. Therefore, the prevalence of FTPI in calves in this study was even higher (42%) [31].

In a New Zealand study of eight farms, the proportion of calves observed suckling at pasture ranged from 40% to 90%. When the calves were measured for IgG after collection from the pasture within 24 h of birth, 64% of calves had suckled successfully from their dams while on pasture [34]. The prevalence of FTPI at day 1 was high though (59%), without additional colostrum feeding [34]. This suggests that the high prevalence of FTPI was a result of the uncontrolled first feeding of calves left with their dams on pasture. Thus, more frequent collection of newborn calves combined with early feeding of colostrum appears advantageous.

#### 4.3.2. Colostrum Quantity and Quality

While most farmers (86%) in Victoria, Australia were reported to feed their calves extra colostrum (1 to 7 L), and 58% of farmers pooled the colostrum from the first milking colostrum of multiple cows, the prevalence of FTPI was still high (38%) [38], implying that both colostrum quantity and quality are crucial even in pasture-based systems. The composition of pooled colostrum is particularly important. In another Australian study, calves fed pooled colostrum at the first feeding had significantly lower FTPI (13%) compared with calves fed a mixture of colostrum and transition milk (55%) [16]. In Manawatu, New Zealand, 100% of farms milked colostrum from the dam to feed their calves at the first feeding, and 73% of farmers indicated that calves received 2 L in the first 6 h and 4 L in the first 12 h. The prevalence of FTPI in these farms was 25% [33].

There were some Australasian dairy farmers who do not provide extra colostrum to the calves. In a study from Victoria, Australia, 14% of farmers did not provide extra colostrum within the first 24 h of calf life and the prevalence of FTPI was 38% as noted above [38]. In New Zealand, 28% of calves were not provided extra colostrum in the first 24 h, and the prevalence of FTPI was 33% [36]. In contrast, another report from New Zealand indicated that when farmers did not provide extra colostrum in the first 24 h of calf life, the FTPI in calves was lower (28%) than on farms where extra colostrum was provided using bottles, feeders or tubes (32%, 35% and 38%, respectively) [36]. The authors suggested that the reason for these results, which conflicted with previous studies, was the colostrum quality [36]. The quality of the extra colostrum offered to calves in this study was extremely poor, with only 10% of 298 tested samples having >22 Brix % and only 9% having bacterial counts below the recommended threshold of 1.00 × 10^5^ cfu/mL [36]. Thus, these results suggest that colostrum quality plays an even more important role in reducing the prevalence of FTPI in calves than the quantity of colostrum.

Colostrum quality can be evaluated via IgG concentration, total coliform count, and total plate count [42]. Assessment of colostrum quality using Brix refractometry to estimate IgG is an easier, faster, and cost-saving method. The accepted threshold of Brix % in colostrum, equivalent 50 g/L of IgG, was estimated to be 22 Brix % [6]. Many farmers in Australasia use Brix % to estimate the colostrum quality before feeding to their calves successfully. For example, farm number 4 in a study by Skirving et al. [16] in South Australia collected newborn calves twice per day and fed calves 2–3 L of colostrum of high quality (>22 Brix %) at the first feed. Consequently, the prevalence of FTPI in calves on this farm was very low (6%). Similarly, In New Zealand, calves had the highest serum total protein and the lowest risk of FTPI if they suckled their dams and then were fed extra colostrum of ≥22% Brix [34].

In addition to IgG concentration in colostrum, the number of bacteria in the colostrum is important for TPI in calves. Bacteria, especially coliforms, may bind free immunoglobulin (Ig) in the gut and block IgG uptake and transport, thereby affecting TPI [6]. In 24 farms in Victoria in Australia, 58% of the colostrum samples met the recommended industry standard of a total plate count of <1.00 × 10^5^ cfu/mL, and 94% of colostrum samples met the recommended industry standard of total coliform count (TCC) of 1.00 × 10^4^ cfu/mL, but only 23% colostrum met all standards [42]. Similarly, another study on farms throughout Australia found that only 20% of the colostrum samples (43/221) met all standards for colostrum quality (IgG, total bacterial count, and total coliform count (TCC)) [31].

## 5. Conclusions

This study highlights the benefits of the “search engine method” over the “traditional search” method for conducting systematic reviews. The meta-analysis based on the literature collected from both search methods indicated that the overall prevalence of FTPI in dairy calves in Australasia was 33% and the prevalence was 38% when individual farm data were analysed. To reduce FTPI in Australia and New Zealand pasture-based systems, it is essential to concentrate on providing adequate colostrum quality and quantity by early separation of calves from their dams, and feeding the newborn calves as soon as possible after birth with first milking pooled colostrum. However, more research on FTPI prevalence in Australasia is necessary before making recommendations for additional management practices locally.

## Figures and Tables

**Table 1:** Keywords used in the systematic search reporting prevalence of failed passive transfer of immunity in dairy calves in Australasia.

	Keywords
1	“passive immunity” AND “Australia”
2	“passive immunity” AND “New Zealand”
3	“immunology” AND “calf” AND “Australia”
4	“immunology” AND “calves” AND “Australia”
5	“immunology” AND “calf” AND “New Zealand”
6	“immunology” AND “calves” AND “New Zealand”
7	“IgG” AND “calf” AND “Australia”
8	“IgG” AND “calves” AND “Australia”
9	“IgG” AND “calf” AND “New Zealand”
10	“IgG” AND “calves” AND “New Zealand”
11	“immunity” AND “transfer” AND “Australia”
12	“immunity” AND “transfer” AND “New Zealand”
13	“immunity” AND “transfer” AND “calf” AND “Australia”
14	“immunity” AND “transfer” AND “calves” AND “Australia”
15	“immunity” AND “transfer” AND “calf” AND “New Zealand”
16	“immunity” AND “transfer” AND “calves” AND “New Zealand”
17	“colostrum” AND “passive immunity transfer” AND “Australia”
18	“colostrum” AND “passive immunity transfer” AND “New Zealand”
19	“colostrum” AND “passive” AND “immunity” AND “transfer” AND “Australia”
20	“colostrum” AND “passive” AND “immunity” AND “transfer” AND “New Zealand”

**Table 2:** Selection criteria for inclusion in the systematic review and meta-analysis of the failed transfer of passive immunity in Australasian dairy calves.

	Criteria
1	Measured transfer of passive immunity (IgG or total protein) in calf serum from 0 to 8 days after birth.
2	Threshold for FTPI was 10 g/L serum IgG or 50–52 g/L serum total protein.
3	Study conducted in Australia or New Zealand.
4	Calves were born on pasture or natural farming.
5	Published, peer-reviewed research article (no proceedings, thesis, newsletter articles, etc.).

**Table 3:** Search results for transfer of passive immunity in calves in Australasia from different sources.

No.	Keywords	Google Scholar ^a^	University Library ^a^	PubMed ^b^	Scopus ^b^	Web of Science ^b^		Total
						Title	Abstract	
1	“passive immunity” AND “Australia”	25	10	8	16	1	6	66
2	“passive immunity” AND “New Zealand”.	18	4	4	6	1	4	37
3	“immunology” AND “calf” AND “Australia”	3	8	1	35	0	1	48
4	“immunology” AND “calves” AND “Australia”	3	10	0	35	0	0	48
5	“immunology” AND “calf” AND “New Zealand”	1	7	0	20	0	0	28
6	“immunology” AND “calves” AND “New Zealand”	5	8	0	20	0	0	33
7	“IgG” AND “calf” AND “Australia”	13	9	2	5	0	2	31
8	“IgG” AND “calves” AND “Australia”	24	9	2	5	0	3	43
9	“IgG” AND “calf” AND “New Zealand”	17	4	6	11	0	2	40
10	“IgG” AND “calves” AND “New Zealand”	29	9	6	11	0	7	62
11	“immunity” AND “transfer” AND “Australia”	11	11	8	37	2	8	77
12	“immunity” AND “transfer” AND “New Zealand”	6	12	12	38	2	10	80
13	“immunity” AND “transfer” AND “calf” AND “Australia”	31	12	4	8	0	4	59
14	“immunity” AND “transfer” AND “calves” AND “Australia”	35	17	5	8	2	4	71
15	“immunity” AND “transfer” AND “calf” AND “New Zealand”	35	9	3	7	0	0	54
16	“immunity” AND “transfer” AND “calves” AND “New Zealand”	40	11	5	7	1	3	67
17	“colostrum” AND “passive immunity transfer” AND “Australia”	23	3	0	2	0	0	28
18	“colostrum” AND “passive immunity transfer” AND “New Zealand”	19	0	0	0	0	0	19
19	“colostrum” AND “passive” AND “immunity” AND “transfer” AND “Australia”	29	11	4	8	0	3	55
20	“colostrum” AND “passive” AND “immunity” AND “transfer” AND “New Zealand”	29	11	4	5	0	1	50
	TOTAL	396	175	74	284	9	58	996

^a^ “Traditional search method”: keywords were searched with “all fields” by using Google Scholar and University of Adelaide library website without accessing a University account. ^b^ “Search engine method”: keywords were searched within “title, abstract and keywords” by using PubMed, Scopus, and Web of Science databases registered via a University of Adelaide library account.

**Table 4:** Description of the eight selected articles for meta-analysis on failed transfer of passive immunity in dairy calves in Australasia.

No.	Article Title	Location	Author	Sample Size	IgG Threshold	TP Threshold	Total Farm	Calf Age
1	An investigation of dairy calf management practices, colostrum quality, failure of transfer of passive immunity, and occurrence of enteropathogens among Australian dairy farms.	Australia	Abuelo et al. [31]	253	10		23	1–7 days
2	Prevalence of failure of passive transfer of immunity in dairy calves in a Mediterranean pasture-based production system of the southwest region of Western Australia.	Western Australia	Aleri et al. [35]	495	10			2–7 days
3	Prevalence and calf-level risk factors for failure of passive transfer in dairy calves in New Zealand.	New Zealand	Cuttance et al. [36]	3819		52	107	1–8 days
4	Prevalence of failure of passive transfer of maternal antibodies in dairy calves in the Manawatu region of New Zealand.	New Zealand	^a^ Lawrence et al. [33]	230		50	11	1–7 days
5	The transfer of passive immunity in calves born at pasture.	New Zealand	^a^ Mason et al. [34]	689		52	8	Day 1 and day 3
6	Prevalence of failure of passive immunity transfer in Australian non-replacement dairy calves.	Victoria	Roadknight et al. [37]	3608		52	956	1–7 days
7	Incidence of inadequate transfer of passive immunity in dairy heifer calves in South Australia.	South Australia	^a^ Skirving et al. [16]	2638		51	5	1–7 days
8	Failure of transfer of passive immunity and agammaglobulinaemia in calves in southwest Victorian dairy herds: prevalence and risk factors.	Victoria	Vogels et al. [38]	1018		50	100	1–7 days

^a^ Articles used for farm level FTPI meta-analysis.

## Data Availability

Data available on request from Thien Van.
